# Facing antimicrobial resistance in cancer care: what the AIOM survey tells us about oncologists’ awareness

**DOI:** 10.1093/jacamr/dlaf150

**Published:** 2025-08-18

**Authors:** A Lasagna, P Cambieri, S Figini, F Baldanti, M Andreoni, F Perrone, N Silvestris, P Pedrazzoli

**Affiliations:** Medical Oncology Unit, Fondazione IRCCS Policlinico San Matteo, Pavia, Italy; Department of Microbiology and Virology, Fondazione IRCCS Policlinico San Matteo, Pavia, Italy; Medical Oncology Unit, Fondazione IRCCS Policlinico San Matteo, Pavia, Italy; Department of Microbiology and Virology, Fondazione IRCCS Policlinico San Matteo, Pavia, Italy; Department of Clinical, Surgical, Diagnostic and Pediatric Sciences, University of Pavia, Pavia, Italy; Department of Systems Medicine, Infectious Disease Clinic, University of Rome Tor Vergata, Lazio, Roma, Italy; Clinical Trial Unit, Istituto Nazionale Tumori, IRCCS Fondazione G.Pascale, Napoli, Italy; Medical Oncology Unit, IRCCS Istituto Tumori ‘Giovanni Paolo II’, Bari 70124, Italy; Medical Oncology Unit, Fondazione IRCCS Policlinico San Matteo, Pavia, Italy; Department of Internal Medicine and Medical Therapy, University of Pavia, Pavia, Italy

## Abstract

**Background:**

Infections with antimicrobial-resistant (AMR) organisms significantly worsen outcomes in cancer patients. Since the main cause of AMR is the inappropriate use of antibiotics for prophylactic or empirical treatment, antimicrobial stewardship programmes (ASPs) have been developed. Few papers on ASPs describe prospective audits in oncology settings. In light of this, the Italian Association of Medical Oncology (AIOM) has decided to evaluate the oncologists’ perceptions of this issue with a brief survey.

**Methods:**

An anonymous 14-item questionnaire was shared online during the XXVI National Congress on the AIOM website. The survey was divided into two sections. The first set of questions collected the hospital organization and practices (Q1–Q6), while the second one was about the clinical practices and antibiotic therapy (Q7–Q14).

**Results:**

Sixty-four medical oncologists completed the anonymous questionnaire. Seventy-two percent of respondents believe that AMR is a significant issue in cancer patients, and an equal percentage considers it likely that a cancer patient will become infected with a MDR pathogen. Despite these concerns, only 48% (*n* = 31/64) report having an active ASP in their centre. Just 6% of respondents (*n* = 4/64) consider themselves very confident in prescribing antibiotic therapy, while most consider themselves fairly or not very confident (*n* = 34/64, 53%, and *n* = 26/64, 36%, respectively).

**Conclusions:**

This survey highlights the urgent need for educating and training oncologists on AMR, as well as the importance of the development of oncology-specific guidelines to mitigate the impact of AMR in this high-risk population.

## Introduction

Patients with cancer are at increased risk for infectious diseases with poorer clinical outcomes compared to immunocompetent subjects.^1^ Infections—primarily of bacterial or fungal origin—contribute to death in up to 50% of patients with solid tumours,^2^ but they can also significantly impair the overall clinical condition. Furthermore, the prolonged use of prophylactic or empirical antibiotics, along with exposure to healthcare-associated infections, predisposes patients with cancer to colonization or infection with antimicrobial-resistant (AMR) organisms.^3^ Raising awareness of AMR among oncologists is essential, as infections caused by MDR microorganisms significantly worsen outcomes in cancer patients.^4^ Since the main cause of AMR is the inappropriate and overuse of antibiotics for prophylactic or empirical treatment, antimicrobial stewardship programmes (ASPs) have been developed to promote the rational use of these therapies.^5^ To date, few papers on ASPs describe prospective audits in oncology settings, because patients with cancer are often excluded from ASP studies.^6,7^ At the global level, leading scientific organizations have initiated training programmes to enhance awareness and education on this topic.^8,9^

In light of this, the Italian Association of Medical Oncology (AIOM) has decided to evaluate the oncologists’ perceptions of this issue through a brief survey to identify potential gaps and inform targeted antimicrobial stewardship interventions in oncology care.

## Methods

### Purpose/aim

The objectives of this study were to assess the level of awareness among Italian oncologists regarding AMR and appropriate antibiotic usage. Additionally, the survey sought to investigate the presence and implementation of ASPs within Italian oncology departments.

### Study design

An anonymous 14-item questionnaire was shared online during the XXVI National Congress on the AIOM website. It was accessible via a direct link sent by e-mail to all congress participants. The survey participants were recruited exclusively during the congress via a voluntary, non-probability sampling method. The survey was divided into two sections (Appendix S1, available as Supplementary data at *JAC-AMR* Online). The first set of questions collected the hospital organization and practices (Q1–Q6), while the second one was about the clinical practices and antibiotic therapy (Q7–Q14). Only closed (multiple-choice, with either single or multiple permitted answers) questions were permitted. The questions (and interpretations in the discussion) were developed in consultation with an infectious disease specialist and were based on the Infectious Diseases Society of America (IDSA) guidelines.^10,11^ Responses considered more or less appropriate reflect current IDSA recommendations, established clinical evidence and standard practice.

### Statistical analysis

Given the descriptive nature of the study and the goal of including all AIOM members, a pre-determined sample size was not established. Descriptive statistics were used to summarize categorical variables, which are reported as absolute numbers and percentages across predefined thematic domains (e.g. hospital organization, clinical practices and AMR awareness).

## Results

### Hospital organization and practices (Q1–Q6)

Between 8 and 10 November 2024, 64 medical oncologists completed the anonymous questionnaire. Ninety-one percent of the respondents were AIOM members. Out of approximately 1000 oncologists attending the AIOM Congress, 64 completed the survey, resulting in an estimated response rate of 6.4%. Only fully completed questionnaires were included in the analysis. The majority of respondents works in public general hospitals with a medical oncology department (*n* = 44/64, 69%). Slightly less than half do not have an Infectious Diseases ward in their hospital (*n* = 29/64, 45%). Thirty-one oncologists (48%) state that their hospital has antimicrobial stewardship (AMS) programme, while the others do not have it and do not know they have it (*n* = 16/64, 25% and *n* = 17/64, 27%, respectively). Oncologists were also asked about the measures their hospital has taken to address AMR. Hospital corporate documents on antibiotic therapy and control of hand gel consumption were found to be the most frequently adopted measures (59% and 41%, respectively). Finally, 47% (*n* = 30/64) of oncologists is used to do rectal screening swabs to all new admissions, while 33% (*n* = 21/64) never does it. Table 1 details these data.

**Table 1.** Hospital organization and practices (Q1–Q6)

Questions	Respondents, *n* (%)
Q1. Are you an AIOM member?	
Yes	58 (91%)
No	6 (9%)
Q2. In which cancer centre do you work?	
Specialized cancer centre	18 (28%)
General hospital with a medical oncology department	44 (69%)
Other	2 (3%)
Q3. Does your hospital have both an oncology and an infectious diseases department?	
Yes	35 (55%)
No	29 (45%)
Q4. Does your hospital have an antimicrobial stewardship (AMS) programme?	
Yes	31 (48%)
No	16 (25%)
Not sure	17 (27%)
Q5. At your hospital, what measures are taken to address the issue of antimicrobial resistance (AMR)? (More than one answer is allowed)	
AMS programme active in all wards	23 (36%)
AMS programme is active only in some wards, *but not* in oncology	8 (13%)
Reporting at set intervals (e.g. monthly) of hospital infections and MDR	24 (38%)
Control of hand gel consumption	26 (41%)
Residential courses on hand washing	25 (39%)
Hospital corporate documents on antibiotic therapy	38 (59%)
Q6. In your oncology department, to which patients are screening rectal swabs administered?	
To all new admissions	30 (47%)
Only to patients undergoing chemotherapy	2 (3%)
Screening rectal swabs are not performed in our department	21 (33%)
We do not have an oncology ward	11 (17%)

### Clinical practices and antibiotic therapy (Q7–Q14)

In this section, we learn that respondents consider AMR a problem for cancer patients, believing that a cancer patient is likely to be infected with an MDR pathogen during hospitalization (*n* = 49/64, 76%). Just 6% of them (*n* = 4/64) consider themselves very confident in prescribing antibiotic therapy, while most consider themselves fairly or not very confident (*n* = 34/64, 53%, and *n* = 26/64, 36%, respectively). Regarding the management of fever, oncologists’ approaches are different: 36% (*n* = 23/64) initiate empirical therapy immediately, 31% (*n* = 20/64) wait 48 h before starting treatment and only 19% (*n* = 12/64) wait for infectious disease consultation. Quinolones and beta-lactams are the preferred antibiotics for empirical therapy (Question 12). Regarding the timing of the reassessment of empirical antibiotic therapy (Question 13), responses varied among oncologists. The largest proportion (44%, *n* = 28/64) reported routinely reassessing treatment after 48 h. A smaller subset (33%, *n* = 21/64) waits for definitive antibiogram results before reconsidering antibiotics, while only 9% (*n* = 6/64) reassess after completing the full 7-day antibiotic course. Notably, 14% (*n* = 9/64) indicated that they do not independently reassess antibiotic therapy, instead relying on infectious disease consultation for treatment evaluation. While recognizing the difficulties in the management of antibiotic therapies, only sometimes they consult microbiologists for antibiogram interpretation (63%, *n* = 40/64). Finally, we asked what oncologists’ approach is to urinary infections, which are one of the most frequent types. The majority of oncologists (66%, *n* = 44/64) only use antibiotic therapy if the infection is symptomatic; however, 14% (*n* = 9/64) always use antibiotic therapy in the case of a positive uroculture, regardless of symptoms or medical history. Table 2 exposes all the responses to the questions about clinical practices and antibiotic therapy.

**Table 2.** Clinical practices and antibiotic therapy (Q7–Q14)

Questions	Respondents, *n* (%)
Q7. In your clinical practice, do you ever consult the microbiology service for interpreting antibiograms?	
Yes, always	8 (13%)
Yes, sometimes	40 (63%)
No, never	16 (25%)
Q8. How confident do you feel in independently prescribing antibiotic therapy?	
Very confident	4 (6%)
Fairly confident	34 (53%)
Slightly confident	26 (36%)
Not at all confident	3 (5%)
Q9. In general, do you think that AMR is a significant problem for cancer patients?	
Yes, I strongly agree	40 (64%)
Yes, but probably more in other types of patients	15 (24%)
No, I do not think it is a significant problem for cancer patients	5 (7%)
Not sure	3 (5%)
Q10. Do you think it is likely that a cancer patient will be infected with an MDR pathogen during hospitalization?	
Yes, I strongly agree	49 (77%)
Yes, but probably more in other types of patients	12 (19%)
No, I do not think it is a significant problem for cancer patients	1 (2%)
Not sure	2 (3%)
Q11. If one of your inpatients develops a fever, what is your course of action?	
I promptly initiate broad-spectrum empirical antibiotic therapy	23 (36%)
I refrain from treating the fever until the results of laboratory tests (humoral and culture) are available	9 (14%)
I wait 48 hours before initiating empirical therapy	20 (31%)
I do not initiate antibiotic therapy independently; I await consultation with an infectious disease specialist	12 (19%)
Q12. When you have to choose an antibiotic for empirical therapy, which class do you tend to prefer?	
Quinolone	31 (48%)
Beta-lactam	25 (39%)
Macrolide	4 (6%)
Trimethoprim/sulfamethoxazole	4 (6%)
Q13. When initiating empirical antibiotic therapy, after what duration do you reassess the treatment?	
After 48 hours, always	28 (44%)
After completing the 7-day antibiotic regimen	6 (9%)
Upon receipt of definitive antibiogram results	21 (33%)
I do not reassess therapy independently; an infectious disease consultation is provided	9 (14%)
Q14. What is your approach in the case of a positive urine culture in an outpatient receiving cancer treatment?	
I always initiate broad-spectrum antibiotic therapy	9 (14%)
I treat only if the patient presents with symptoms	42 (66%)
I treat with antibiotics only if the patient is neutropenic	8 (13%)
I treat only if nitrites and esterase are detected on urine analysis, regardless of symptoms or medical history	5 (8%)

## Discussion

This survey provides valuable insights into the current awareness and practices of Italian oncologists regarding AMR and antibiotic use in oncology. The results, while limited by low participation in the survey, highlight several critical issues that need to be addressed.

Firstly, although the majority of respondents recognize AMR as a significant concern for cancer patients, this awareness does not consistently translate into correct and standardized clinical practice. The Lancet series on ‘Sustainable Access to Effective Antibiotics’ has vigorously stated that the rising number of MDR pathogenic infections globally is a primary issue that must be addressed through appropriate use of antibiotic therapies and by adopting effective preventive measures.^12^ Also in oncological settings, MDR organisms can be a serious issue. For example, in Norway, cancer patients appear to have a higher risk of acquiring vancomycin-resistant enterococci, which is predominantly healthcare-associated, and a lower risk of methicillin-resistant *Staphylococcus aureus* (MRSA), as MRSA is often linked to community exposure or international travel, which patients with cancer may limit during treatment.^13^ Raising oncologists’ awareness of AMR is crucial, as infections with MDR organisms dramatically compromise the prognosis of cancer patients. Shelke *et al.*^4^ reported that infections caused by MDR or XDR pathogens, in patients with solid tumours, were associated with longer hospital stays—18.30 ± 11.14 days and 22.83 ± 13.22 days, respectively—compared to infections caused by bacteria sensitive to all antibiotics (16.90 ± 10.23 days).

Most oncologists reported low to moderate confidence in prescribing antibiotic therapy. This lack of confidence probably reflects the limited infectious disease support in many oncology departments, as almost half of the respondents reported the absence of a dedicated infectious disease unit in their hospitals. A significant percentage of oncologists are unaware of active ASPs in their departments. This finding aligns with some reports indicating that oncology departments were excluded from the implementation of ASPs. The limited integration of ASPs in oncology units may partially explain the variability in empirical treatment strategies and antibiotic re-evaluation times reported in the survey. Few papers investigated ASPs in the management of infections among patients with cancer.^14–16^ Raheem *et al.*^14^ showed that pharmacist-led ASPs were effective in reducing rates of antibiotic resistance, even in outpatient settings of patients with cancer. Mohammed *et al.*^15^ developed and validated a questionnaire designed to assess pharmacists’ knowledge and practices related to antimicrobial stewardship in oncology care, demonstrating strong psychometric properties and reliability. Itoh *et al.*^16^ demonstrated that the introduction of ASPs led to a significant reduction in carbapenem days of therapy (CAR-DOT) per 100 patient-days per month, alongside a reduction in the length of hospital stay. Collectively, these studies contribute important insights into ASPs relevant to oncology care.

Regarding infection control practices, institutional measures, such as hand hygiene monitoring and antibiotic policy documents, have been implemented inconsistently. Our finding that nearly half of the respondents reported universal rectal screening should be interpreted with caution. This variability in screening practices reflects differences in institutional protocols and highlights the need for standardized guidelines tailored to oncology settings.

The wide variation in the timing of initiation and re-evaluation of antibiotic therapies, the unnecessary antibiotic exposure in the case of asymptomatic bacteriuria, and the reliance on external consultation rather than independent clinical judgement also emphasize the need for targeted training and guidelines adapted to oncology settings.

The predominant use of quinolones and beta-lactams for empirical treatment is a reflection of common practice, but raises concerns given the increasing resistance to these agents in nosocomial pathogens.^17,18^

This work has several limitations that need to be acknowledged. Firstly, the survey was completed by only 64 oncologists, which may not fully represent the population of Italian oncologists. The limited sample size is a common issue in many surveys, including recent ones.^19,20^ Furthermore, the sample was non-random and self-selected, as participants were recruited during a national oncology congress. This setting may have led to an overrepresentation of academically engaged or AMR-aware clinicians, further reducing the generalizability of the results to the broader population of Italian oncologists. Secondly, the answers are self-reported responses, which are subject to recall bias and social desirability bias. Respondents may have over- or under-reported certain practices or perceptions. Moreover, the survey’s introductory wording may have primed participants, potentially influencing their responses and introducing response bias. Due to the exploratory and descriptive design of this study, statistical correlations between oncologists’ awareness, confidence in antibiotic prescribing and their clinical practices were not assessed.

Despite these limitations, this study presents several noteworthy strengths. In particular, our paper is timely and unique, addressing a critical and underexplored issue—the awareness and practices related to AMR among oncologists—at a time when AMR represents a growing issue for immunocompromised populations, including patients with cancer. Additionally, although the sample size is very small, the survey included oncologists from various types of hospitals throughout Italy, providing a preliminary but valuable snapshot of national practices related to AMR and antibiotic use (and abuse) in oncology.

### Conclusions

In summary, our survey emphasizes the need for increased integration of ASPs within oncology departments, with particular focus on education and multidisciplinary collaboration with infectious disease specialists and clinical microbiologists in routine cancer care. Finally, the development of oncology-specific guidelines might support more rational antibiotic use and help mitigate the impact of AMR in this high-risk population.

## Supplementary Material

dlaf150_Supplementary_Data

## Data Availability

Obtainable after reasonable request.

## Supplementary data

Appendix S1 is available as Supplementary data at *JAC-AMR* Online.

## References

[1] Lasagna A . What is the oncologist’s role in vaccination? Future Oncol 2024; 20: 1451–4. 10.1080/14796694.2024.2355081 38861310 PMC11441023

[2] Boire A, Burke K, Cox TR et al Why do patients with cancer die? Nat Rev Cancer 2024; 24: 578–89. 10.1038/s41568-024-00708-4 38898221 PMC7616303

[3] Nanayakkara AK, Boucher HW, Fowler VG Jr et al Antibiotic resistance in the patient with cancer: escalating challenges and paths forward. CA Cancer J Clin 2021; 71: 488–504. 10.3322/caac.21697 34546590

[4] Shelke A, Priya P, Mishra S et al Investigation of antimicrobial susceptibility patterns, risk factors and their impact on mortality in cancer patients at a tertiary care cancer hospital—a prospective study. Ann Clin Microbiol Antimicrob 2024; 23: 59. 10.1186/s12941-024-00703-5 38926734 PMC11210011

[5] Barlam TF, Cosgrove SE, Abbo LM et al Implementing an antibiotic stewardship program: guidelines by the Infectious Diseases Society of America and the Society for Healthcare Epidemiology of America. Clin Infect Dis 2016; 62: e51–77. 10.1093/cid/ciw118 27080992 PMC5006285

[6] Aitken SL, Nagel JL, Abbo L et al Antimicrobial stewardship in cancer patients: the time is now. J Natl Compr Canc Netw 2019; 17: 772–5. 10.6004/jnccn.2019.7318 31319394

[7] Tverdek FP, Aitken SL, Mulanovich VE et al Implementation of an automated antibiotic time-out at a comprehensive cancer center. Open Forum Infect Dis 2024; 11: ofae235. 10.1093/ofid/ofae235 38798895 PMC11127483

[8] Lasagna A, Cambieri P, Baldanti F et al How should we manage the impact of antimicrobial resistance in patients with cancer? An oncological and infectious disease specialist point of view. JCO Oncol Pract 2025: OP2400935. 10.1200/OP-24-00935 39977722

[9] Elkrief A, Routy B, Derosa L et al Gut microbiota in immuno-oncology: a practical guide for medical oncologists with a focus on antibiotics stewardship. Am Soc Clin Oncol Educ Book 2025; 45: e472902. 10.1200/EDBK-25-472902 40262063

[10] Taplitz RA, Kennedy EB, Bow EJ et al Antimicrobial prophylaxis for adult patients with cancer-related immunosuppression: ASCO and IDSA clinical practice guideline update. J Clin Oncol 2018; 36: 3043–54. 10.1200/JCO.18.00374 30179565

[11] Taplitz RA, Kennedy EB, Bow EJ et al Outpatient management of fever and neutropenia in adults treated for malignancy: American Society of Clinical Oncology and Infectious Diseases Society of America clinical practice guideline update. J Clin Oncol 2018; 36: 1443–53. 10.1200/JCO.2017.77.6211 29461916

[12] https://www.thelancet.com/pb/assets/raw/Lancet/stories/series/antimicrobial-resistance/amr-series-exec-summ2024.pdf

[13] Danielsen AS, Elstrøm P, Eriksen-Volle H-M et al The epidemiology of multidrug-resistant organisms in persons diagnosed with cancer in Norway, 2008–2018: expanding surveillance using existing laboratory and register data. Eur J Clin Microbiol Infect Dis 2024; 43: 121–32. 10.1007/s10096-023-04698-3 37980302 PMC10774199

[14] Raheem M, Anwaar S, Aziz Z et al Adherence to the core elements of outpatient antibiotic stewardship: a cross-sectional survey in the tertiary care hospitals of Punjab, Pakistan. Infect Drug Resist 2020; 13: 3833–41. 10.2147/IDR.S268574 33149628 PMC7602895

[15] Mohammed AH, Hassan BAR, Sern LJ et al Development and validation of a questionnaire assessing pharmacists’ knowledge and practice towards antimicrobial stewardship in oncology care. PLoS One 2025; 20: e0321551. 10.1371/journal.pone.0321551 40408398 PMC12101630

[16] Itoh N, Akazawa-Kai N, Yamaguchi M et al Efficiency of a long-term infectious diseases consultation and antimicrobial stewardship program at a Japanese Cancer Center: an interrupted time-series analysis. Open Forum Infect Dis 2024; 11: ofae678. 10.1093/ofid/ofae678 39605975 PMC11597401

[17] Sharma V, Saini M, Das R et al Recent updates on antibacterial quinolones: green synthesis, mode of interaction and structure–activity relationship. Chem Biodivers 2025; 22: e202401936. 10.1002/cbdv.202401936 39756027

[18] Kherroubi L, Bacon J, Rahman KM. Navigating fluoroquinolone resistance in Gram-negative bacteria: a comprehensive evaluation. JAC Antimicrob Resist 2024; 6: dlae127. 10.1093/jacamr/dlae127 39144447 PMC11323783

[19] Lasagna A, Brunello A, Silvestris N et al Italian oncologists and vaccinations against infectious diseases: results of a survey of the Italian Association of Medical Oncology. Tumori 2024; 110: 60–8. 10.1177/03008916231191547 37586016 PMC10851644

[20] Zagami P, Esposito A, Taurelli Salimbeni B, et al Advancing treatment choices: CDK4/6 inhibitor switching in HR+/HER2− metastatic breast cancer. Breast. 2025; 79: 103875. 10.1016/j.breast.2025.103875. 39826385 PMC11786079

