# Supplementary material for: Facing antimicrobial resistance in cancer care: what the AIOM survey tells us about oncologists’ awareness
Source: JAC Antimicrob Resist. 2025 Aug 18;7(4):dlaf150. doi: 10.1093/jacamr/dlaf150 (PMC12359028; doi:10.1093/jacamr/dlaf150)
Supplement: dlaf150_Supplementary_Data [file dlaf150_supplementary_data.docx]

**National survey on the perception and attitudes of Italian physicians towards *antimicrobial resistance* in patients with cancer**

**Dear colleague, cancer patients have a 3 times higher risk of dying from an infection than a patient without cancer. AntiMicrobial Resistance (AMR) increases the frequency of sepsis, the length of hospitalization, sepsis-related mortality, and it is a major public health problem.**

**We therefore propose this short survey to focus on the real perception of the problem by oncologists.**

Q1. Are you an AIOM member?

- Yes
- No

Q2. In which kind of oncological centre do you work?

- Specialized dedicated anticancer Center
- General hospital with oncology unit
- Other

Q3. Does your hospital have both an Oncology and an Infectious Diseases department?

- Yes
- No

Q4. Does your hospital have an antimicrobial stewardship (AMS) programme?

- Yes
- No
- I don't know

Q5. In your clinical practice, do you ever contact the Microbiology Service for antibiogram interpretation?

- Yes, always
- Yes, sometimes
- No, never

Q6. In your hospital, what measures are taken to reduce the problem of AMR? (*More than one answer is allowed*)

- AMS programme active in all wards
- AMS programme active only in some wards, BUT NOT in oncology ward
- Reporting at set intervals (e.g. monthly) of hospital infections and MDR
- Control of hand gel consumption
- Residential courses on hand washing
- Company documents on antibiotic therapy

Q7. How confident do you feel in prescribing antibiotic therapy autonomously?

- Very confident
- Fairly confident
- Slightly confident
- Not at all confident
- Q8. In general, do you think that AMR is a significant problem in cancer patients?
- Yes, I strongly agree
- Yes, but it is probably more in other types of patients
- No, I do not think it is a significant problem in cancer patients
- I don't know

Q9. Do you think it is likely that a cancer patient will be infected with an MDR pathogen during hospitalisation?

- Yes, I strongly agree
- Yes, but probably more in other types of patients
- No, I do not think it is a significant problem in cancer patients
- I don't know

Q10. If one of your in-patients develops fever, what do you do:

- I immediately start broad-spectrum empirical therapy
- I do not treat the fever until the results of biohumoral and culture tests
- I wait 48 hours before setting empirical therapy
- I do not start antibiotic therapy on my own, only after infectivological consultation

Q11. When you have to choose an antibiotic for empirical therapy, which class do you tend to prefer?

- Quinolone
- Beta-lactam
- Macrolide
- Trimethoprim/sulfamethoxazole

Q12. When you start empirical antibiotic therapy, after how long do you re-evaluate the therapy?

- After 48 hours, always
- After completion of the 7-day antibiotic course
- With definitive antibiogram results
- I do not re-evaluate therapy, an infectious counselling service is provided

Q13. What do you do in the case of a positive uroculture in an outpatient undergoing cancer treatment?

- I always treat with broad-spectrum antibiotic therapy
- Treat only in case of symptoms
- Treat with antibiotic therapy only if the patient is neutropenic
- Treat only if nitrites and esterase are present on urine examination, regardless of symptoms and anamnesis

Q14. In your oncology department to whom are screening rectal swabs given?

- To all new admissions

- Only patients undergoing chemotherapy

- Screening rectal swabs are not done in our department

- We do not have an oncology inpatient ward
